# Editorial: Advances in immunotherapy of hepatic-biliary-pancreatic cancers, volume II

**DOI:** 10.3389/fonc.2024.1537218

**Published:** 2025-01-27

**Authors:** Hu Jiaming, Zhang Minghui, Liu Huiqiang, Zhang Zongli, Xu Yunfei

**Affiliations:** Department of General Surgery, Qilu Hospital of Shandong University, Jinan, China

**Keywords:** immunotherapy, immune checkpoint inhibitors, tyrosine kinase inhibitor, hepatocellular carcinoma, biliary tract cancer

Hepatic and bile duct cancers are one of the deadliest cancers, and their incidence rate and mortality rate are rising in recent years. Radical surgical resection is the best way to treat early hepatocellular carcinoma (HCC) and biliary tract cancers (BTCs), however, there is no obvious clinical symptoms in the early stages. For later stage unresectable patients, chemotherapy, targeted therapy and immunotherapy are optional and effective treatments.

In this Research Topic, there are four articles, including two case reports and two reviews, have been published. These articles mainly report or summarize the clinical practice of immunotherapy and(or) targeted therapy in Hepatic and bile duct cancers.

Liang et al. presented one case of unresectable Sarcomatoid hepatocellular carcinoma (SHC) where surgical resection was possible after combined treatment with lenvatinib and camrelizumab; the combined approach showed excellent therapeutic effect. The authors provide a compelling narrative of how immunotherapy and targeted therapy can work synergistically to alter tumor biology, potentially converting unresectable cases into operable ones. This report also offers a valuable literature review of treatment approaches for SHC, emphasizing the need for further clinical validation of combination therapies in this context. By demonstrating the feasibility and success of surgery post-systemic therapy, the study paves the way for future research into improving outcomes in patients with this challenging malignancy.

Abraham and Samson presents the first documented use of atezolizumab and bevacizumab combination therapy in a patient with multifocal hepatocellular carcinoma (HCC) undergoing hemodialysis. It was shown that, despite the dual challenges of advanced liver cancer and end-stage renal disease (ESRD), the patient achieved partial tumor response and stable disease over two years, with manageable toxicity levels. The authors highlight the feasibility of this therapeutic approach, emphasizing its potential to balance efficacy and safety in this vulnerable patient population. Notably, the report discusses the interplay of immune checkpoint inhibitors (ICIs) with VEGF-targeted therapy in modulating the tumor microenvironment, while addressing unique complications such as wound infections and renal toxicities. This study underlines the necessity of multidisciplinary care, particularly between oncology and nephrology teams, to optimize outcomes for ESRD patients with HCC and calls for further research into tailored treatments for such complex cases.

Although significant achievements have brought to some cancer patients, they tend to show various response and have different effects after accept ICIs treatments. There is a huge need to explore predictive biomarkers to optimize patient selection to maximize efficacy and minimize toxicities. Qin et al. categorized systematically biomarkers of ICIs treatment into biochemical, tumor-related, and immune-related factors, as well as imaging and personal characteristics etiology, gut microbiome, and immune-related adverse events (irAEs). While established biomarkers like AFP levels, ALBI scores, and CRAFITY scores are showing potential, emerging indicators such as gut microbiota, circulating tumor cells (CTCs), and artificial intelligence-driven analyses are paving the way for novel strategies. Qin et al. highlights both the promise and challenges of integrating predictive biomarkers into clinical workflows, underscoring the importance of dynamic and robust biomarker monitoring to refine patient selection and maximize therapeutic outcomes with ICIs in HCC.

Yoon et al. comprehensively evaluates the role of PD-L1 expression as a predictive biomarker for anti-PD-1/PD-L1 therapy in biliary tract cancer (BTC). While PD-L1 expression showed no significant correlation with objective response rate (ORR) or disease control rate (DCR), it was associated with improved progression-free survival (PFS) and overall survival (OS), highlighting its prognostic rather than predictive utility. The authors delve into the complexities of PD-L1’s role, noting how treatment modalities, such as monotherapy versus combination therapy, and diagnostic cut-off levels significantly influence its predictive value. This study underscores the need for standardization in PD-L1 assessment methods and calls for further investigation into other complementary biomarkers, such as tumor mutational burden and microsatellite instability, to enhance patient stratification. By addressing the current gaps and limitations in biomarker research, this work advances the understanding of PD-L1’s role in BTC immunotherapy and lays the groundwork for improving therapeutic decision-making in this aggressive malignancy.

